# Developing a theoretical model and questionnaire survey instrument to measure the success of electronic health records in residential aged care

**DOI:** 10.1371/journal.pone.0190749

**Published:** 2018-01-09

**Authors:** Ping Yu, Siyu Qian

**Affiliations:** Centre for IT-enabled Transformation, School of Computing and Information Technology, Faculty of Engineering and Information Sciences, University of Wollongong, Wollongong, New South Wales, Australia; Nederlands Instituut voor Onderzoek van de Gezondheidszorg, NETHERLANDS

## Abstract

Electronic health records (EHR) are introduced into healthcare organizations worldwide to improve patient safety, healthcare quality and efficiency. A rigorous evaluation of this technology is important to reduce potential negative effects on patient and staff, to provide decision makers with accurate information for system improvement and to ensure return on investment. Therefore, this study develops a theoretical model and questionnaire survey instrument to assess the success of organizational EHR in routine use from the viewpoint of nursing staff in residential aged care homes. The proposed research model incorporates six variables in the reformulated DeLone and McLean information systems success model: system quality, information quality, service quality, use, user satisfaction and net benefits. Two variables training and self-efficacy were also incorporated into the model. A questionnaire survey instrument was designed to measure the eight variables in the model. After a pilot test, the measurement scale was used to collect data from 243 nursing staff members in 10 residential aged care homes belonging to three management groups in Australia. Partial least squares path modeling was conducted to validate the model. The validated EHR systems success model predicts the impact of the four antecedent variables—training, self-efficacy, system quality and information quality—on the net benefits, the indicator of EHR systems success, through the intermittent variables use and user satisfaction. A 24-item measurement scale was developed to quantitatively evaluate the performance of an EHR system. The parsimonious EHR systems success model and the measurement scale can be used to benchmark EHR systems success across organizations and units and over time.

## Introduction

According to the International Organization for Standardization, electronic health records (EHR) are “repository of patient data in digital form, stored and exchanged securely, and accessible by multiple authorized users. It contains retrospective, concurrent, and prospective information and its primary purpose is to support continuing, efficient and quality integrated healthcare” [1].

Electronic health record systems are increasingly introduced into various healthcare organizations worldwide to improve quality and safety for patient care, financial and operational efficiency for organizations [2] and societal benefits (e.g. improved population health and reduced costs). Given the broad scope and decisive role in influencing every aspect of health care, EHR projects can absorb a significant amount of funding and take long time to establish [3,4]. Implementing it is also a challenge [1,4,5,6], facing considerable obstacles, such as the unintended negative consequences [7] and end user resistance [8]. Hence, it is very important to develop a theoretical model and a questionnaire survey instrument to measure end user perceptions about EHR implementation success, particularly impact on quality and safety of patient care. This useful information can support the decision makers to develop timely, targeted interventions to address challenges, avoid resistance and ensure implementation success.

### Theoretical base

The theoretical base of this study is DeLone and McLean’s (D&M) information systems (IS) success model. This model provides a comprehensive understanding of IS success by identifying and explaining the relationships of six critical variables for IS success. These variables are system quality, information quality, IS use, user satisfaction, individual impact and organizational impact [9]. In 2003, DeLone and McLean updated their model to include an independent variable service quality. All the ‘impact’ variables were grouped into a single impact variable, ‘net benefits’, a generalized term that encompasses all levels and types of impacts of IS, including individual, work group, organizational, inter-organizational, consumer and societal impacts [10].

### Prior efforts of applying D&M IS success model to measure health information system success using questionnaire survey method

To date, only a few studies have used the D&M IS success model, or the modified quantitative predictive model, as a theoretical framework to guide the design of a questionnaire survey study that evaluates EHR system effectiveness [5,11,12,13]. The reliability and validity of these studies is undermined for various reasons. For example, Bossen et al. did not formally validate the survey instrument [11]. Otieno et al. did not test the relationship among the variables in the model [5]. Messeri et al. did not include information quality into their model; the reliability of the construct ease of use was also poor [13]. Garcia-Smith and Effken only included four variables in their model [12]. They used a two-stage approach to test the regression model. As the relationship between the primary independent variables and the third stage dependent variable ‘net benefit’ was not directly tested, the reliability of the relationship was undermined.

Given the prominence of EHR investment around the world and the paucity of comprehensive, parsimonious theoretical models and easy-to-use questionnaire survey instrument to assess EHR performance, this study aims to develop and validate an integrated EHR systems success model based on the D&M IS success model. The research aims are (1) to develop an EHR success model; (2) to develop and validate a questionnaire survey instrument that can empirically test and theorize the model; and (3) to examine the associations among the variables and their relative impact on EHR systems success.

### Research model and hypotheses

Eight variables are tested in our model: training, self-efficacy, system quality, information quality, service quality, use, user satisfaction and net benefits. The definition of each variable in this study, its measurement and proposed relationship with the other variables is given below.

#### Training

‘Training is the organized activity aimed at imparting information and/or instructions to improve the recipient’s performance or to help him or her attain a required level of knowledge or skill’ [14]. Yaghmaie and Jayasuriya suggest that health staff with better computer training have more positive attitudes toward computers, less computer anxiety and more awareness of others’ expectations about computer use than untrained staff [15]. Many aged care staff members have little computer knowledge or documentation capability [16] and in Australia more than 90% of them are female [14]. Our discussion with care staff members also suggested that their perception of the system were highly influenced by the level of training and support services they received [2]. Training is therefore included in our model as a distinct variable.

#### Self-efficacy

Self-efficacy is conceptualized as one’s belief in his or her own capacity to use an EHR system, in analogy with the well-established definition of computer self-efficacy [17]. As nursing staff often rely on training and peer support to learn how to use an EHR system [14]; therefore we propose:

H1: Training (a) predicts nursing staff’s self-efficacy to use an EHR system.

#### System quality

System quality is a system’s overall performance, as perceived by users [10]. It measures technical success of an EHR system. The main measurement items are ease of use, usefulness, ease of learning, etc. [18].

#### Information quality

Information quality is the desirable characteristics of the system output, such as outcome reports [18]. It represents the semantic success of an EHR system. A total of 49 attributes are identified [19]. The major ones include relevance, accuracy, understandability, etc.

#### Service quality

According to Petter et al., service quality refers to the quality of the support that system-users receive from the IS department and support personnel [18]. The attributes include dependability, availability and empathy of the support staff.

#### Use

Use is the degree and manner in which staff and customers utilize the capabilities of an IS [18], e.g. amount, frequency, and extent of use. Doll and Torkzadeh advocate that system use is an appropriate measure of success in most cases and is a key variable in understanding IS success [20] because an IS will bring in net benefits only when it is adequately used [21]. DeLone and McLean posit that system quality, information quality and service quality predict use [10]. Self-efficacy is also an important factor determining end user’s use of IS [22]. Therefore, it is posited:

H2: Self-efficacy (a), system quality (b), information quality (c) and service quality (d) predict use.

#### User satisfaction

User satisfaction is users’ level of overall satisfaction with their interaction with an IS [18]. Because satisfaction reflects the utility of the IS in decision making for end-users, it is hard to deny the success of a system which users like [21]. Therefore, satisfaction is regarded as the most common measure of IS success [23]. DeLone and McLean suggest that system quality, information quality, service quality and use positively impact on user satisfaction [9]. Therefore, it is hypothesized:

H3: System quality (a), information quality (b), service quality (c) and use (d) predict user satisfaction with an EHR system.

#### Net benefits

Net benefits are the degree to which a user believes that using a system will result in benefits such as an increase in job performance or productivity to the user or the organization [24]. The term net benefits in this study denotes the positive impacts of the EHR systems on residents, care staff and aged care organizations that have introduced the systems. DeLone and McLean suggest user satisfaction will positively predict net benefits; therefore, it is posited that:

H4: Use (a) and user satisfaction (b) predict net benefits of an EHR system.

**Fig 1:**
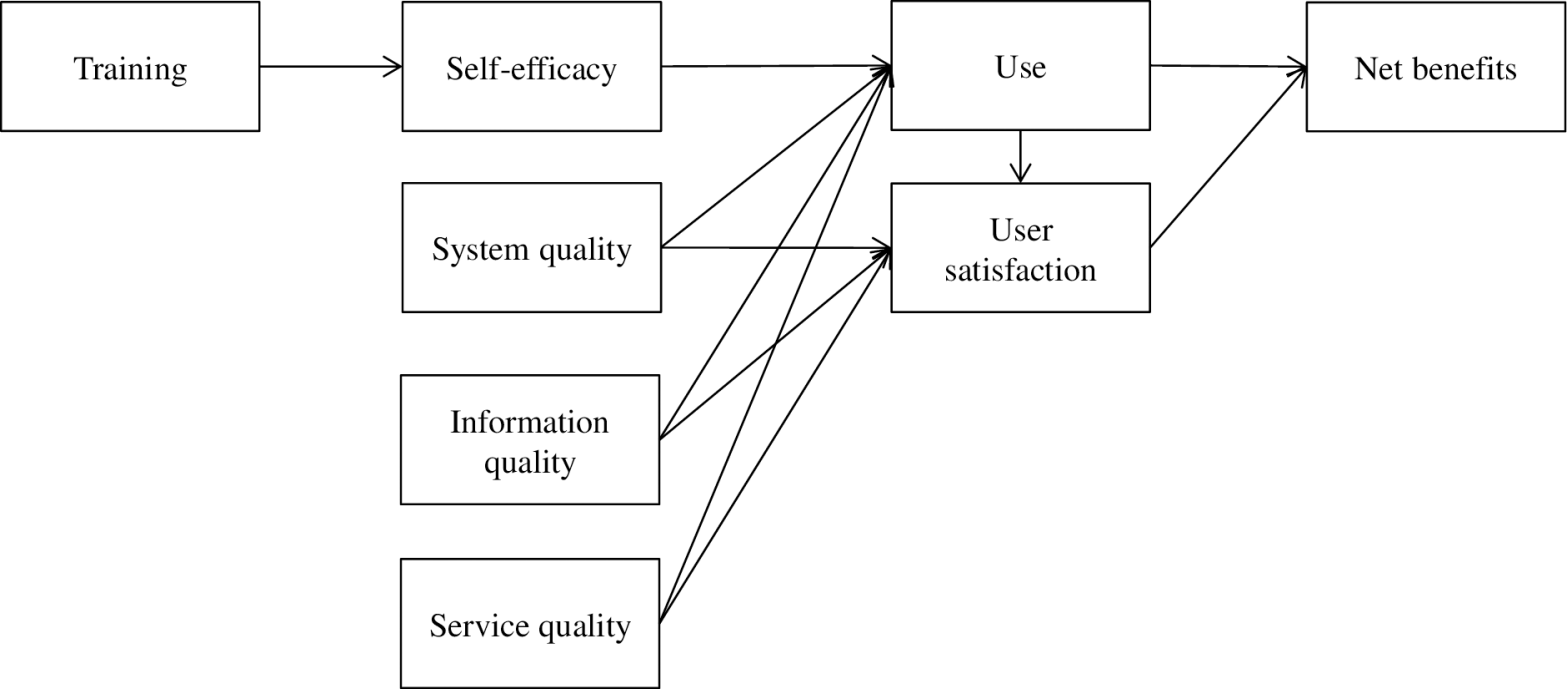
The hypothesized EHR systems success model.

The hypothesized model is presented in Fig 1. Table 1 summarizes the study hypotheses.

**Table 1:** The research hypotheses of this study.

H1: Training (a) predicts nursing staff’s self-efficacy to use an EHR system.
H2: Self-efficacy (a), system quality (b), information quality (c) and service quality (d) predict use.
H3: System quality (a), information quality (b), service quality (c) and use (d) predict user satisfaction with an EHR system.
H4: Use (a) and user satisfaction (b) predicts net benefits of an EHR system.

## Methods

### Ethics approval

The study was approved by the Human Research Ethics Committee, University of Wollongong and Uniting. The written permission for the survey was acquired from the aged care organizations RSL Care, Uniting and Warrigal Care, who had entered a formal research partnership with the University of Wollongong. Written consent was obtained from the participants.

### The EHR systems to be evaluated

Documentation in residential aged care in Australia (equivalent to long-term care or nursing homes in other countries) is mandatory according to the government accreditation and funding requirements. Residential aged care in Australia is in the initial stages of introducing EHR to improve resident care quality, efficiency and regulatory compliance. In this study, two commercial EHR systems, one Web-based, one desktop but run on a Microsoft.NET framework, were evaluated. The systems were designed to automatically integrate data captured on forms, charts and progress notes into nursing care plans, calculation of funding and generation of clinical management reports [25]. The functions of the systems included residents’ demographic information, admission and ongoing assessment of health history, care plans, progress notes, residents’ forms and charts, incident and accident reports [25]. Both systems automatically generated reports. System 2 also contained administrative and 24-h shift handover reports.

### The implementation context

The two systems were implemented in 10 residential aged care homes belonging to three not-for-profit organizations in three states of Australia. These aged care homes provided both personal care and nursing care to the older people, with sizes ranging from 20 to 160 beds. System 1 was introduced in two aged care homes belonging to Organization 1 in Queensland state in 2005 to 2006. The system had been used for four to five years by the time of survey. System 2 was implemented in 2007 to 2008 in eight aged care homes belonging to Organizations 2 and 3 in New South Wales and Australia Capital Territory. The system was in use for 2 to 3 years by the time of the survey.

The EHR systems had been used by all categories of nursing staff members. Personal care workers or assistants in nursing entered progress notes and incident reports into the EHR systems and read information about a resident when the need arose, most often on a daily basis. Enrolled nurses or endorsed enrolled nurses assessed residents’ health conditions and entered data into the EHR systems, in addition to daily progress reporting. Registered nurses were responsible for recording everything about a resident, including assessment, care plan, progress reports, incident reports, etc. The administrative staff and nursing managers also used the system for tracking residents’ information when the need arose. Visiting health professionals, such as general practitioners, physiotherapists, podiatrists, were given the opportunity to use the system to read and enter data to share with the aged care homes.

### Survey participants

Survey participants were all types of care staff members working in the aged care homes. These included facility managers, registered nurses, enrolled nurses, endorsed enrolled nurses, personal care workers and recreational officers. Due to difficulty of access, visiting health professionals were not included in the study.

### Instrument development

A Likert scale self-administered questionnaire was used to collect data to measure the eight latent variables and test the theoretical relationships among them (see S1 Appendix and S2 Appendix). The questionnaire was composed of two parts. The first part consisted of 24 questions that measured seven of the eight variables in the research model. Except use, each measurement item was assessed on a 7-point Likert scale, anchored between 1-‘strongly disagree’ to 7-‘strongly agree’. To avoid forcing a response decision, the option ‘not applicable’ was provided.

Three items were developed to measure use: Item 1 was worded as ‘How many minutes per shift do you spend on the system?’ Item 2 was ‘How many times a shift do you log on to the system?’ Item 3 was ‘How many functions in the system have you used?’ Nine major functions were listed for the participants to choose. The total number of functions selected was counted.

To ensure reliability, the original questionnaire items were adopted from previous validated studies, modified to fit with our study context. Training was measured by three items from Yaghmaie and Jayasuriya [15]. Self-efficacy was measured by two items adopted from Venkatesh et al. [26]. System quality was measured by three items adopted from Kline [27] and another item adopted from consultation with an aged care expert. Information quality consisted of four items from Hartman et al. [28]. Service quality was measured by three items from Kline [27]. Use was measured by one item from Henry and Stone [29], with two items added after discussion with the domain experts and field observation of nurses interaction with the systems. User satisfaction was gauged by seven items from Hartman et al. [28]. One item was used to measure overall satisfaction. Net benefits were tested by seven items from Mairinger et al. [30].

Recognizing the importance of domain context in defining and measuring each variable of IS success [10,31], a pre-test was conducted through discussion with 24 domain experts, including five RNs, eight aged care nursing managers, three Chief Executive Officers of aged care organizations, three employees of a health IT technology company and five information managers in public health organizations. The resulting instrument was highly specific to the aged care context. The instrument was then further validated in two aged care homes, with results published in [16,25].

The second part of the questionnaire elicited respondent demographics, including gender, age, job role, employment status, shifts worked and length of work in the current aged care home.

### Field study sites and data collection

Exploratory cross-sectional data collection was conducted between January and April 2011. Convenience sampling was used for recruiting survey participants. There were two channels for distribution of the 374 copies of the questionnaire: (1) distributed face-to-face by the researchers to the participants during site visits and immediately collected and (2) distributed by the facility management. In this case, an envelope was attached to the questionnaire together with an information sheet and the consent form in order to ensure informed consent and anonymity of responses for this channel of distribution. Reminder calls were made one week later to remind the facility management to collect responses. A period of two to three weeks was given for the administration. A small incentive program of free entry to a raffle draw to win grocery shopping tickets was given to the survey participants in each aged care home. 243 copies of questionnaires were returned, a response rate of 65%.

### Data processing and analysis

To make optimal use of the valuable observed data, mean imputation method was used to replace a missing value with the average value of a variable [32]. Structural equation modeling was then applied to test the measurement model, i.e. the relationships within the variables, and the structural model, i.e. the hypothesized relationships simultaneously [33].

Structural equation modeling was conducted using partial least squares path modeling [34] in open source software package R Version 3.4.0 [35]. The indicators with the loadings lower than the recommended value and the path coefficients which were not significant were iteratively deleted from the model. The path coefficients for the trimmed model were calculated and tested. The significance of the correlations between the latent variables was tested in IBM SPSS version 19. The significance level was set at p < 0.05.

The reliability and validity of a measurement model is assessed by its psychometric properties. The psychometric properties of the model are assessed by internal consistency, convergent and discriminant validity.

For reflective indicators, internal consistency is measured by composite reliability [36], with the recommended acceptable value of 0.70 [37]. System quality and information quality were viewed as the effect of the indicators rather than the causes of them, thus they had formative indicators and were irrelevant in assessment of the internal consistency [38]. Convergent validity is measured by average variance extracted (AVE). It is adequate when each variable has an AVE of at least 0.50 [39]. Discriminant validity is the extent to which a variable is truly distinct from other variables [36]. It is acceptable if the square root of the AVE of each variable is greater than the correlation between this variable and the other variables in the model. Discriminant validity is also tested by the loadings and cross loadings. The loading of an indicator on its assigned variable should be higher than its cross loadings on all the other variables.

A structural model includes the unobservable latent variables and the theoretical relationships among them [39]. It suggests how well the theoretical model predicts the hypothesized paths or relationships.

A sensitivity analysis did not find any significant differences in the mean values for seven constructs between System 1 and System 2 except the construct of use (Mean value for System 1: 3.37, mean value for System 2: 4.09, p < 0.001). As System 2 had more functions than System 1, it was reasonable for it to be more used. We also tested the model with or without the data from System 1. Little change was found in two models; therefore, it was appropriate to combine the data collected from the two systems to increase the representativeness of the study.

## Results

### Characteristics of the participating nursing staff

Overall, 73.7% of the respondents were personal care workers or assistants in nursing and all had the same level of education, i.e. Aged Care Certificate III or IV from the registered training organisations such as the Technical and Further Education College in Australia. Registered nurses with university nursing degrees accounted for 9.9% of the participants. This was in accordance with the national census data [40]. The other characteristics of the participating nursing staff captured were gender, age, organization, employment status, shift and length of work in their aged care homes (see Table 2).

**Table 2:** The demographic information of the participating nursing staff.

Characteristics	Frequency (%)
Gender	
Male	25 (10.3)
Female	218 (89.7)
Age	
Under 20	3 (1.2)
20–30	33 (13.6)
31–40	76 (31.3)
41–50	76 (31.3)
51–60	1 (0.4)
above 60	13 (5.3)
No answer	41 (16.9)
Job role	
Personal care workers/Assistant in nursing/ Recreational officer	179 (73.7)
Endorsed enrolled nurse/ Enrolled nurse	16 (6.6)
Registered nurse	24 (9.9)
Manager / Director of Nursing	11 (4.5)
Other	3 (1.2)
No answer	10 (4.1)
Organization working for	
Organization 1	27 (11.1)
Organization 2	145 (59.7)
Organization 3	71 (29.2)
Employment status	
Full time	59 (24.3)
Part time	145 (59.7)
Casual	35 (14.4)
No answer	4 (1.6)
Shift to work	
Morning	146 (60.1)
Afternoon	63 (25.9)
Night	27 (11.1)
Rostering	3 (1.2)
No answer	4 (1.6)
Length of work in their aged care homes	
Less than 3 months	2 (0.8)
3 months to 1 year	41 (16.9)
1 to 5 years	98 (40.3)
More than 5 years	102 (42.0)

Similar to the national data [40], approximately 90% of the survey respondents were female. 46.1% of nursing staff members were under 40 years old. 31.7% were between the age of 40 to 60 years and only 5.3% were over 60 years old.

### Descriptive statistics of the theoretical variables

As shown in Table 3, the scores of the means for all of the latent variables except use were positive (close to or more than 5 in 7 Likert scale), suggesting that the users responded favorably to the EHR systems introduced. All variables had significant positive correlations with each other.

**Table 3:** Number of indicators, mode, mean and standard deviation (SD) of latent variables, composite reliability (CR) and average variance extracted (AVE), and correlations between latent variables.

Latent variables	No.	Mode	Mean	SD	CR	AVE	1	2	3	4	5	6	7
1. Training	3	Reflective	5.12	1.47	0.90	0.75	0.87						
2. Self-efficacy	2	Reflective	5.91	1.32	0.97	0.94	0.58	0.97					
3. System quality	4	Formative	5.64	1.29	0	0	0.69	0.73	0				
4. Information quality	4	Formative	5.73	1.20	0	0	0.65	0.68	0.86	0			
5. Use	3	Reflective	4.01	1.28	0.88	0.72	0.28	0.35	0.19	0.19	0.85		
6. User satisfaction	1	Reflective	5.57	1.57	1	1	0.59	0.59	0.82	0.81	0.06	1	
7. Net benefits	7	Reflective	5.03	1.39	0.92	0.61	0.61	0.53	0.68	0.69	0.22	0.64	0.78

The matrix diagonal presents the square roots of the AVEs.

Correlation coefficients ranged from 0.06 to 0.86. Strong correlations were found for information quality and system quality (0.86), user satisfaction and system quality (0.82), user satisfaction and information quality (0.81), system quality and self-efficacy (0.73). Interestingly, all weak correlations were between use and other variables (0.06 to 0.35).

### The measurement model

As shown in Table 3, the values of the composite reliability of the seven latent variables ranged from 0.88 to 1, which is above the recommended acceptable value of 0.70 [37]. The AVE of the variables ranged from 0.61 to 1 (excluding the two variables with formative indicators). This confirmed that these variables were valid in representing distinct variables. As user satisfaction was only measured by one item, its AVE was 1.The square roots of the AVEs, presented in the matrix diagonal, were greater in all cases than the off-diagonal elements in their corresponding column. Again, system quality and information quality were excluded for being formative indicators.

As shown in Table 4, the loadings of all the 24 items were significant, all exceeding 0.70. The loading of an indicator on its assigned variable was higher than its cross loadings on all the other variables. Therefore, discriminant validity was validated by both loadings and cross loadings.

**Table 4:** Weights, loadings and cross loadings of the model.

Latent variables and indicators	Weight	Loadings and cross loadings						
		1	2	3	4	5	6	7
1. Training								
Tr1	0.45	**0.89**	0.58	0.63	0.59	0.30	0.54	0.53
Tr2	0.28	**0.78**	0.36	0.49	0.49	0.18	0.44	0.51
Tr3	0.42	**0.93**	0.54	0.64	0.60	0.23	0.55	0.57
2. Self-efficacy								
SE1	0.52	0.56	**0.97**	0.68	0.63	0.35	0.54	0.52
SE2	0.51	0.57	**0.97**	0.73	0.68	0.32	0.60	0.51
3. System quality								
SysQ1	0.18	0.54	0.65	**0.84**	0.72	0.16	0.69	0.59
SysQ2	0.26	0.55	0.63	**0.85**	0.73	0.14	0.70	0.60
SysQ3	0.17	0.66	0.65	**0.78**	0.71	0.23	0.63	0.59
SysQ4	0.54	0.62	0.63	**0.92**	0.78	0.16	0.76	0.59
4. Information quality								
IQ1	0.07	0.55	0.63	0.69	**0.73**	0.31	0.59	0.59
IQ2	0.17	0.48	0.53	0.62	**0.69**	0.25	0.55	0.56
IQ3	0.16	0.60	0.67	0.78	**0.86**	0.21	0.69	0.63
IQ4	0.71	0.61	0.61	0.81	**0.97**	0.13	0.78	0.63
5. Use								
U1	0.43	0.25	0.30	0.19	0.21	**0.88**	0.07	0.21
U2	0.37	0.21	0.28	0.15	0.15	**0.82**	0.04	0.18
U3	0.38	0.25	0.30	0.15	0.13	**0.85**	0.03	0.16
6. User satisfaction								
US1	1.00	0.59	0.59	0.82	0.81	0.06	**1.00**	0.64
7. Net benefits								
NB1	0.21	0.53	0.44	0.53	0.55	0.23	0.55	**0.78**
NB2	0.22	0.56	0.47	0.64	0.61	0.13	0.60	**0.78**
NB3	0.15	0.34	0.33	0.42	0.46	0.19	0.39	**0.76**
NB4	0.19	0.48	0.49	0.60	0.57	0.15	0.50	**0.82**
NB5	0.17	0.40	0.41	0.48	0.49	0.23	0.43	**0.79**
NB6	0.16	0.48	0.35	0.44	0.51	0.20	0.41	**0.77**
NB7	0.19	0.50	0.37	0.53	0.54	0.05	0.54	**0.75**

### The structural model

**Fig 2:**
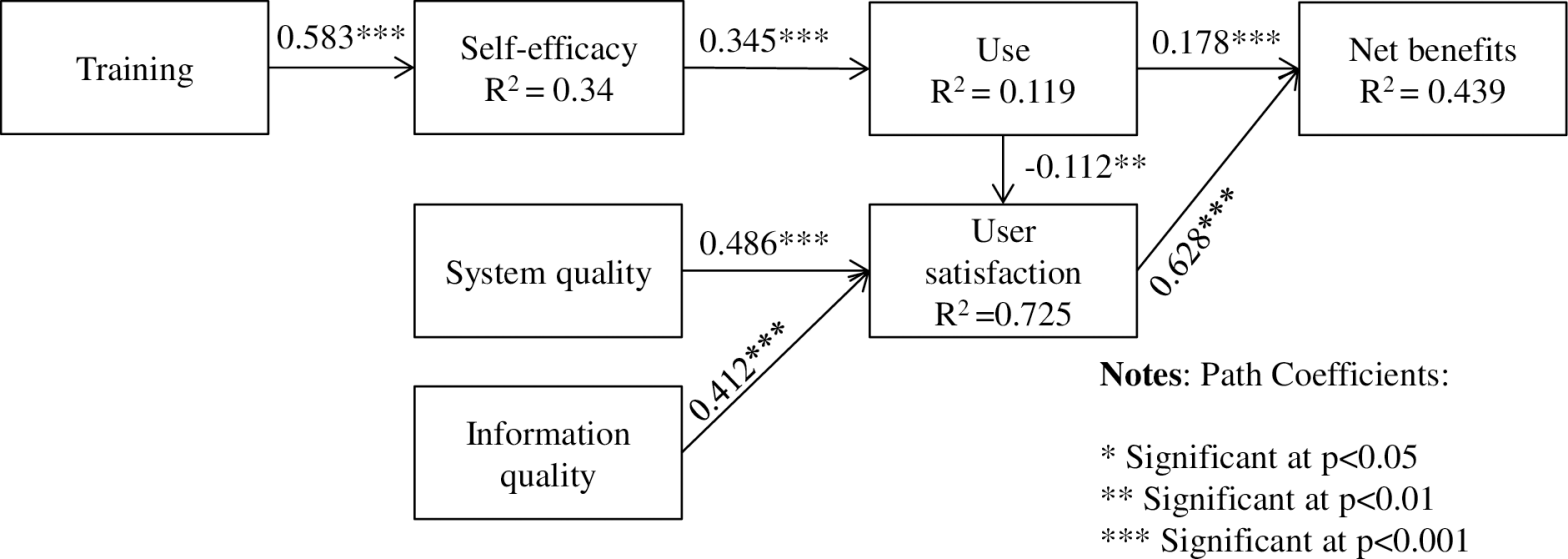
The structural model.

Fig 2 shows the validated structural model, with the values of the path coefficients and variance explained (R^2^ value) presented. The path coefficients suggest the strength of the relationships between the variables [34]. The R^2^ value indicates the percentage of variance predicted in the model. All path coefficients were positive except the path from use to user satisfaction being negative.

Table 5 presents the hypotheses that were supported by the analysis results. In contrast to the original hypotheses, service quality was excluded from the model. System quality and information quality did not have any direct effect on use. The direct, indirect and total effects were shown in Table 6.

**Table 5:** The research hypotheses that are supported.

Hypotheses supported
H1: Training (a) predicts self-efficacy.
H2: Self-efficacy (a) predicts use.
H3: System quality (a), information quality (b) and use (c) predict user satisfaction.
H4: Use (a) and user satisfaction (b) predict net benefits.

**Table 6:** The direct, indirect and total effects of antecedent and dependent variables on the other dependent variables.

Relationships (A predicts B)		Direct	Indirect	Total
A	B			
Training	Self efficacy	0.58	0	0.58
Self efficacy	Use	0.35	0	0.35
Training	Use	0	0.20	0.20
System quality	User satisfaction	0.49	0	0.49
Information quality	User satisfaction	0.41	0	0.41
Training	User satisfaction	0	-0.02	-0.02
Self efficacy	User satisfaction	0	-0.04	-0.04
Use	User satisfaction	-0.11	0	-0.11
User satisfaction	Net benefits	0.63	0	0.63
System quality	Net benefits	0	0.30	0.30
Information quality	Net benefits	0	0.26	0.26
Use	Net benefits	0.18	-0.07	0.11
Self efficacy	Net benefits	0	0.04	0.04
Training	Net benefits	0	0.02	0.02

## Discussion

This empirical study developed an EHR success model (Fig 2) and measurement scale (see S4 Appendix and S5 Appendix) to assess the ongoing performance of EHR in Australian aged care setting at routine usage stage. Seven out of 11 original hypotheses about the relationships among seven variables were supported (see Tables 1 and 5). It leads support to the impact of four antecedent variables—training, self-efficacy, system quality and information quality—on the net benefits, the indicator of EHR systems success, through the intermittent variables use and user satisfaction.

As the two EHR systems had been used for five and three years, respectively, in the relevant workplace, they had formed their independent evaluation of the system after extensive, long-term usage of it in daily work practice. Therefore, their usage and perceived benefits of the system appeared not to be influenced by the support services provided by their organization anymore. Hypotheses 2d and 3c were not supported.

The above-average, positive value of each variable suggests that the EHR systems had performed very well according to the evaluation of the direct users, the nursing staff. At the conceptual level, the quantitative model was also supported by content analysis of the qualitative data collected in the same population [2].

The following sections will discuss the rationale for the selection of research methods, the relationships among the variables, the challenges for measurement, and the limitations of the study.

### The advantage of structural equation model over multiple regression in multivariate, multi-level theoretical model development

In the traditional multivariate regression model, a dependent variable is predicted by one or more antecedent variables. The relationship between the antecedent and the dependent variables is fixed, which works for simple scenarios with very few factors. However, in a complex scenario, the relationship between the antecedent and the dependent variables can be dynamic. For example, in our model, the variable use is the dependent variable for self-efficacy, it is also the antecedent variable for user satisfaction and net benefits. These complex models compose a Structural Equation Model (SEM) [41]. Comparing with separately fitted regression models, the advantage of SEM is transparent. It simultaneously estimates the multiple relationships of each variable, jointly and comprehensively, to reflect the entire structural or hierarchical relations in the data.

### System quality and its measurement

Petter and Fruhling measured system quality by nine items, including ease of use, accessibility and speed [42]. Garcia-Smith and Effken measured system performance by three items, ease of use, access and reliability [12]. We measure system quality by four items, usefulness, ease of use, easy to learn and retrieve information easily. As the meaning of our measurement items are not interchangeable, we measured this construct formatively [43]. Factor analysis and SEM confirmed the validity of our measurement scale.

### Training, self-efficacy, use and user satisfaction

As computers were not widely used in Australia until the 1980s, it is not likely that the 37% of the respondents over 40 years of age received formal computer training during their school education. This fact supports the importance of training for improving nursing staff’s self-efficacy of using the EHR. As found, self-efficacy explained 12% of the variance in use. Therefore, inclusion of the variables training and self-efficacy in the model adds knowledge about the factors influencing nursing staff’s self-efficacy to use the EHR system.

Interestingly, the impact of the variable use to the output variable user satisfaction was negative. This may suggest that the more the nursing staff used the EHR, the less satisfied they were with the system.

### The relationship between the three antecedent variables—system quality, information quality and service quality—and use

A previous study did not find any relationship between system quality, information quality and service quality and use of an emergency response medical information system. The authors interpreted it as a lack of need to use the system unless an emergency arose [42]. The same result was replicated in our study. The three antecedent variables—system quality, information quality and service quality—had no significant impact on use. Hypotheses 2b, 2c and 2d were all rejected. Although the EHR system was used on a regular basis by nursing staff and managers for data entry and retrieval, they only used it when need arose. The reason may be that the nature of mandatory use had made the nursing staff felt obliged to use the system no matter which level of system quality, information quality or support they received. This may also explain the weak correlation between use and the other variables. Therefore, the validated model can be used to predict or assess the performance of EHR in routine use instead of the original one.

### Challenges for measuring use

Use has often been measured as actual use, self-reported use, depth of use, and importance of use [18]. Each attempt of operationalization is addressing different aspects of the use construct, which is inconsistent. Several researchers have highlighted the weakness in measuring use [18,31,44], or overlooking use. For example, Szajna did not believe perceived use to be an appropriate surrogate for actual use [45] on the ground that users are often poor estimators of aspects of their own behavior [46]; therefore, Devaraj and Kohli recommend that IT impacts should best be assessed by examining actual IT use rather than self-reported use [47]. DeLone and McLean suggest that the measurement of use should reflect the nature, extent, quality and appropriateness of system use [10]. Seddon and Kiew recommend that when use is compulsory, the amount of time a system is used does not directly relate to usefulness or success [23,31], whereas perceived usefulness may be a more meaningful success construct. Other researchers also suggest that non-use does not necessarily mean that a system is not useful; it may simply be because the potential users have other tasks to do and could not spend more time using the system [10,23,31]. Thus Petter and McLean suggest that use should be based on needs, not only amount and frequency [48]; a view shared by some personal care workers and managers in this study. We, therefore, share the view that a reasonable measure of use needs to be further developed to capture the richness and full functionality of an EHR system.

### The relationship between use and user satisfaction

After reviewing 26 studies that examined the relationship between use and user satisfaction, Petter et al. believe that the relationship between use and user satisfaction has been consistently weak [18], a view that is supported by the finding of this study. Gelderman also find that the association between use and net benefits was not statistically significant. What they believe is that use was necessary but not sufficient to create system benefits [49]. Contrary to the finding of Gelderman [49], a weak, yet significant relationship between use and net benefits was established in this study. What is interesting is the relationship between use and user satisfaction was negative, suggesting that the more the end users used the system, the less satisfied they were with it.

### The relationship of user satisfaction and information quality or net benefits

Seddon and Kiew find that system quality and information quality are significant determinants of overall user satisfaction for an EHR [50]. We adopted Doll and Torkzadeh’s end-user computing satisfaction scale to measure satisfaction [51]. This scale conceptualized satisfaction as a collection of beliefs about the information provided by an IS. It was overlapped in semantics with the scale measuring information quality. To avoid multi-collinearity, only a single global item “Overall, I am satisfied with the EHR system” was finally integrated into the model, a sub-optimal option, although was also used by Otieno et al. [5] and Mairinger et al. [30]. Despite user satisfaction being well explained (73%) by the three variables information quality, system quality and use, its measurement could be further improved.

In the future, the semantic differential technique to measure satisfaction adopted by Bhattacherjee [52] and Venkatesh et al. [53] along bipolar evaluative dimensions (e.g., good/bad) [54] could be adopted to improve the measurement of satisfaction. Another option is to adopt one item from Petter and Fruhling’s instrument, “I like having the STATPack^TM^ system available” [42] and modify one item from the instrument of Messeri et al. “I would recommend our current EHR to other colleagues” [13].

44% of variations in net benefits are predicted by user satisfaction and use, with user satisfaction possessing 63% of direct effect. This supports the observation that user satisfaction is the best prediction of IS success [55].

### Limitations

This study is, obviously, limited by its empirical scope and geographic location, and the EHR application that the nursing staff were introduced to use. There is an inability to link input variables to the real health care outcomes of the older people [13] nor nursing work efficiency. These limitations were partially addressed by taking other research approaches, such as auditing national aged care accreditation reports about residential aged care services [56] and observational study about nurses’ interaction with the EHR system [57].

Another limitation was the choice of constructs, which was based on our preliminary research and literature study, therefore, can be further improved. Several IS studies have observed discrepancies between perceived and actual performance; therefore, other methods of investigation are needed to triangulate the findings from different channels.

It is likely that the performance of each variable and their indicators may change over time with changes in any conditions at the study context; however, our predictive model should remain due to the application of the robust structural equation modeling method to generate it.

There are statistical limitations associated with survey sampling. The measurement for satisfaction could include more items. The results can be affected by non-response bias, which could not be measured due to the anonymous nature of the survey. The participant demographic profiles are similar among the three participant organizations, as well as coincide with the care staff profiles suggested by a recent national survey [40]; therefore, sampling bias is unlikely.

Another limitation of the study is not using control variables. All of the organizations participating in the study were non-profit, medium to large size aged care organizations in Australia. The organizational type, size and culture could potentially influence the dependent variables. This limitation suggests that a future direction of the research would be to replicate the study in different health care worker populations, health care settings and countries.

Generalizability of the study was guarded by the respondents including 243 nursing staff using two EHR systems from 10 residential aged care homes in three organizations spreading over three states in Australia. However, no single study can provide a complete assessment of the performance of a measurement scale; therefore the psychometric properties of the instrument need to be verified in any further studies that apply our instrument in other environments with other health information systems and types of users. The EHR success model can also be improved through fine-tuning the measurement items and the inclusion of more variables. For example, although self-efficacy is integrated into the model, compatibility and facilitators, which were found to have significant impact on healthcare providers’ intention to use telemedicine solutions [58], can be examined as well.

Comparison of different levels and types of nursing staff members’ perceptions about the EHR performance in different organizations may shed further light on the impact of environmental factors on end user perceptions of EHR systems success. Another direction is to measure EHR success at aged care facility or organizational level, linking the input variables to objective output variables such as quality of records, organizational performance [57] and patient care outcomes [13].

## Conclusion

This study developed and tested a theoretical model and questionnaire survey instrument to measure EHR systems success. It tested the mutual influences among variables: training to self-efficacy, self-efficacy to use, system quality, information quality and use to user satisfaction, and finally use and user satisfaction to net benefits of an EHR system. The validated EHR systems success model and measurement scale are useful for the evaluation and auditing of routine use and management of EHR systems on a formative as well as summative basis. This will identify areas that have improved or need further improvement. The approach and constructs can be referenced by other organizational health IT projects. The findings will also enrich the body of knowledge of IS effectiveness measurement.

### Implications for practice

The validated EHR systems success model and measurement scale can be used by EHR evaluators, organizational decision makers and system implementers to predict the success of their EHR initiatives, to assess the need for improving system and end user training, and to identify the healthcare workers who may hold suboptimal view about any one of the seven dimensions of EHR use determinants. These would be useful for the design and implementation of the relevant interventions such as system upgrade, further training for end users to improve their comfort to use the system and quality of information captured in the system.

## Supporting information

S1 Appendix Original survey questionnaire. (DOC)

S2 Appendix Original measurement items. (DOCX)

S3 Appendix Original dataset. (CSV)

S4 Appendix Refined survey questionnaire. (DOC)

S5 Appendix Refined measurement items. (DOCX)

S6 Appendix Refined dataset. (CSV)
