# Supplementary material for: Developing a theoretical model and questionnaire survey instrument to measure the success of electronic health records in residential aged care
Source: PLoS One. 2018 Jan 9;13(1):e0190749. doi: 10.1371/journal.pone.0190749 (PMC5760016; doi:10.1371/journal.pone.0190749)
Supplement: S1 Appendix — (DOC) [file pone.0190749.s001.doc]

**S1 Appendix. Original survey questionnaire**

### Electronic health record system usage survey

Please circle the number on the descriptive scale based on your own experiences and feelings. There are no good or bad answers.

|  | Strongly disagree | Disagree | Slightly disagree | Neutral | Slightly agree | Agree | Strongly agree | Don’t know |
| --- | --- | --- | --- | --- | --- | --- | --- | --- |
| 1. The electronic health record system is easy to use. | 1 | 2 | 3 | 4 | 5 | 6 | 7 | 0 |
| 2. The electronic health record system is useful. | 1 | 2 | 3 | 4 | 5 | 6 | 7 | 0 |
| 3. The electronic health record system is easy to learn. | 1 | 2 | 3 | 4 | 5 | 6 | 7 | 0 |
| 4. Information from the system is relevant to my work. | 1 | 2 | 3 | 4 | 5 | 6 | 7 | 0 |
| 5. Information I get from the system is accurate. | 1 | 2 | 3 | 4 | 5 | 6 | 7 | 0 |
| 6. It is easy to understand information from the system. | 1 | 2 | 3 | 4 | 5 | 6 | 7 | 0 |
| 7. The information is presented in a useful format. | 1 | 2 | 3 | 4 | 5 | 6 | 7 | 0 |
| 8. I can retrieve information I need easily. | 1 | 2 | 3 | 4 | 5 | 6 | 7 | 0 |
| 9. Overall, I am satisfied with the electronic health record system. | 1 | 2 | 3 | 4 | 5 | 6 | 7 | 0 |
| 10. When I enter data into the computer, I feel confident about what I am doing. | 1 | 2 | 3 | 4 | 5 | 6 | 7 | 0 |
| 11. I feel comfortable to use the electronic health record system. | 1 | 2 | 3 | 4 | 5 | 6 | 7 | 0 |
| 12. The support services for the system are dependable. | 1 | 2 | 3 | 4 | 5 | 6 | 7 | 0 |
| 13. The support services give me individual attention. | 1 | 2 | 3 | 4 | 5 | 6 | 7 | 0 |
| 14. Overall, the support services meet my needs. | 1 | 2 | 3 | 4 | 5 | 6 | 7 | 0 |
| 15. There was enough time for me to familiarise with the system. | 1 | 2 | 3 | 4 | 5 | 6 | 7 | 0 |
| 16. I have access to ongoing training. | 1 | 2 | 3 | 4 | 5 | 6 | 7 | 0 |
| 17. The training I received was relevant to how I should use the system. | 1 | 2 | 3 | 4 | 5 | 6 | 7 | 0 |
| 18. Using the system has helped me to manage resident’s funding. | 1 | 2 | 3 | 4 | 5 | 6 | 7 | 0 |
| 19. Using the system has helped me to manage resident’s care. | 1 | 2 | 3 | 4 | 5 | 6 | 7 | 0 |
| 20. Has improved communication with other health service providers (e.g. GPs). | 1 | 2 | 3 | 4 | 5 | 6 | 7 | 0 |
| 21. Has facilitated me to exchange care strategies with co-workers. | 1 | 2 | 3 | 4 | 5 | 6 | 7 | 0 |
| 22. Has facilitated the identification of trends and patterns. | 1 | 2 | 3 | 4 | 5 | 6 | 7 | 0 |
| 23. Has facilitated the development of care plans. | 1 | 2 | 3 | 4 | 5 | 6 | 7 | 0 |
| 24. Gives me useful reminders that help me to identify the change of care needs for a resident in a timely manner. | 1 | 2 | 3 | 4 | 5 | 6 | 7 | 0 |

**Basic Demographics and System Usage**

1. Gender:  Female  Male

2. Your age:  Under 20 yrs  20–30 yrs  31–40 yrs  41-50 yrs  51-60 yrs  above 60 yrs

3. You are employed as:

 Personal care workers/Assistant in nursing/ Recreational officer

 Endorsed enrolled nurse/Enrolled nurse  Registered nurse  Manager/Director of Nursing

 Other, please specify­­­­­­­­­­­____________________

4. Your work:  Full time  Part time  Casual

5. Which shift do you work on the day of answering the question?

 Morning  Afternoon  Night  Rostering

6. How long have you worked in this facility?

 Less than 3 months  3 months to 1 year  1 to 5 years  More than 5 years

7. How many minutes per shift do you spend on the system?

 None  Less than 5 min.  5 to 10 min.  10 to 20 min.

 20 to 30 min.  30 to 60 min.  61 to 120 min.  2 to 4 hours

8. How many times a shift do you log on to the system?

 None  Once  2 to 9 times  10 to 19 times  More than 20 times

9. Which functions in the system have you used?

 Progress notes

 Care plans

 Assessment forms

 Charts

 Upload photos

 Handover sheet

 Resident details

 Management reports

 Printing
