# Supplementary material for: Developing a theoretical model and questionnaire survey instrument to measure the success of electronic health records in residential aged care
Source: PLoS One. 2018 Jan 9;13(1):e0190749. doi: 10.1371/journal.pone.0190749 (PMC5760016; doi:10.1371/journal.pone.0190749)
Supplement: S2 Appendix — (DOCX) [file pone.0190749.s002.docx]

**S2 Appendix. Original measurement items**

*Training*

Tr1. There was enough time for me to familiarize with the system.

Tr2. I have access to ongoing training.

Tr3. The training I received was relevant to how I should use the system.

*Self-efficacy*

SE1. When I enter data into the computer, I feel confident about what I am doing.

SE2. I feel comfortable to use the system.

*System quality*

SysQ1. The system is easy to use.

SysQ2. The system is useful.

SysQ3. The system is easy to learn.

SysQ4. I can retrieve information I need easily.

*Information quality*

IQ1. Information from the system is relevant to my work.

IQ2. Information I get from the system is accurate.

IQ3. It is easy to understand information from the system.

IQ4. The information is presented in a useful format.

*Service quality*

SerQ1. The support services for the system are dependable.

SerQ2. The support services give me individual attention.

SerQ3. Overall, the support services meet my needs.

*Use*

U1. How many minutes per shift do you spend on the system?

U2. How many times a shift do you log on to the system?

U3. How many functions in the system have you used?

*User satisfaction*

US1. Overall, I am satisfied with the system.

*Net benefits*

NB1. Using the system has helped me to manage resident’s funding.

NB2. Using the system has helped me to manage resident’s care.

NB3. Has improved communication with other health service providers (e.g. GPs).

NB4. Has facilitated me to exchange care strategies with co-workers.

NB5. Has facilitated the identification of trends and patterns.

NB6. Has facilitated the development of care plans.

NB7. Gives me useful reminders that help me to identify the change of care needs for a resident in a timely manner.
